# Effects of *Bacillus subtilis* or *Lentilactobacillus buchneri* on aerobic stability, and the microbial community in aerobic exposure of whole plant corn silage

**DOI:** 10.3389/fmicb.2023.1177031

**Published:** 2023-04-17

**Authors:** Hang Yin, Meirong Zhao, Gang Pan, Hongyu Zhang, Rui Yang, Juanjuan Sun, Zhu Yu, Chunsheng Bai, Yanlin Xue

**Affiliations:** ^1^College of Horticulture, Shenyang Agricultural University, Shenyang, China; ^2^Institute of Grassland Research, Chinese Academy of Agricultural Sciences, Hohhot, China; ^3^College of Grassland Science and Technology, China Agricultural University, Beijing, China; ^4^Inner Mongolia Engineering Research Center of Development and Utilization of Microbial Resources in Silage, Inner Mongolia Academy of Agricultural and Animal Husbandry Science, Hohhot, China; ^5^Inner Mongolia Key Laboratory of Microbial Ecology of Silage, Inner Mongolia Academy of Agricultural and Animal Husbandry Science, Hohhot, China

**Keywords:** silage, aerobic exposure, fermentation quality, bacterial community, fungal community

## Abstract

This study aimed to evaluate the effects of *Bacillus subtilis* or *Lentilactobacillus buchneri* on the fermentation quality, aerobic stability, and bacterial and fungal communities of whole plant corn silage during aerobic exposure. Whole plant corn was harvested at the wax maturity stage, which chopped to a length of approximately 1 cm, and treated with the following: distilled sterile water control, 2.0 × 10^5^ CFU/g of *Lentilactobacillus buchneri* (LB) or 2.0 × 10^5^ CFU/g of *Bacillus subtilis* (BS) for 42 days silage. Then, the samples were exposed to air (23–28^°^C) after opening and sampled at 0, 18 and 60 h, to investigate fermentation quality, bacterial and fungal communities, and aerobic stability. Inoculation with LB or BS increased the pH value, acetic acid, and ammonia nitrogen content of silage (*P* < 0.05), but it was still far below the threshold of inferior silage, the yield of ethanol was reduced (*P* < 0.05), and satisfactory fermentation quality was achieved. With the extension of the aerobic exposure time, inoculation with LB or BS prolonged the aerobic stabilization time of silage, attenuated the trend of pH increase during aerobic exposure, and increased the residues of lactic acid and acetic acid. The bacterial and fungal alpha diversity indices gradually declined, and the relative abundance of *Basidiomycota* and *Kazachstania* gradually increased. The relative abundance of *Weissella* and *unclassified_f_Enterobacteria* was higher and the relative abundance of *Kazachstania* was lower after inoculation with BS compared to the CK group. According to the correlation analysis, *Bacillus* and *Kazachstania* are bacteria and fungi that are more closely related to aerobic spoilage and inoculation with LB or BS could inhibit spoilage. The FUNGuild predictive analysis indicated that the higher relative abundance of fungal parasite-undefined saprotroph in the LB or BS groups at AS2, may account for its good aerobic stability. In conclusion, silage inoculated with LB or BS had better fermentation quality and improved aerobic stability by effectively inhibiting the microorganisms that induce aerobic spoilage.

## 1. Introduction

Whole plant corn silage is a high-quality source of roughage and is widely used in ruminant diets (Gallo et al., 2022) because of the rich nutrition and high feeding value that can effectively meet the production requirements of ruminants (Chen et al., 2014; Cui et al., 2022). However, whole plant corn silage has high levels of residual soluble carbohydrates and lactic acid, and low concentrations of natural antifungal compounds, making it more prone to aerobic spoilage after opening during production (Lara et al., 2016; Kung et al., 2018). Thus, improvement in the aerobic stability of corn silage is a conventional but constant concern for making high-quality and safe silage (Huang et al., 2021).

Aerobic spoilage is caused by the growth of aerobic microorganisms such as yeasts and filamentous fungi, which can metabolize lactic acid under aerobic conditions, thereby leading to its deterioration (Mendonça et al., 2020; Ferrero et al., 2021a). The use of microbial inoculants, such as lactic acid bacteria (LAB), is an effective means of reducing aerobic spoilage and the accumulation of toxic matter during the ensiling process (Bernardi et al., 2019; Liu et al., 2020). LAB fermentation types are divided into homofermentative and heterofermentative types, *Lentilactobacillus buchneri* is the most commonly used heterofermentative bacterial species (Benjamim da Silva et al., 2021); it is capable of producing a mix of lactic and acetic acids and has been used to improve the aerobic stability of silages (Basso et al., 2012; Mangwe et al., 2016). The addition of *L. buchneri* to silage improved aerobic stability by fermenting lactic acid to acetic acid, which inhibits filamentous fungi growth, thereby prolonging the duration that silage can be exposed to air and slowing its deterioration (Kung and Ranjit, 2001; Zhang et al., 2021). However, the heterofermentative pathway is less efficient in terms of both acidification and preservation of nutrients during the anaerobic storage phase than the homofermentative pathway (Wilkinson and Davies, 2013; Guan et al., 2021). Therefore, our goal is search for silage inoculants that can not only ensure the quality of silage fermentation, but also improve aerobic stability.

*Bacillus subtilis* can inhibit the growth of pathogenic bacteria and strengthen intestinal barrier function, which is beneficial to ruminant growth performance, and has a good capacity for ruminants to maintain the microbial balance of the gastrointestinal tract (Zhang et al., 2017). Studies have confirmed that inoculation of *B. subtilis* in silage can improve silage fermentation (Guo et al., 2022), by producing cellulases, converting water soluble carbohydrates into lactic acid and decreasing pH, thereby inhibiting harmful microbial activity, and has the potential to be a silage inoculant (Li et al., 2018). In addition, researchers have confirmed that *B. subtilis* can produce antifungal and antibacterial substances, that can inhibit the growth of undesirable microorganisms such as yeast and filamentous fungi, and prevent silage spoilage and mycotoxin production (Bonaldi et al., 2021), which may be applicable to improving aerobic stability. Inoculation with *B. subtilis* can improve the aerobic stability of silage (Bumbieris et al., 2017; Bai et al., 2020), but studies on its role in whole plant corn silage are inadequate. Gandra et al. (2017) observed that co-inoculation of *L. buchneri* and *B. subtilis* improved the aerobic stability of sunflower silage by reducing the number of aerobic and spoilage microorganisms and increasing the number of lactic acid bacteria. However, the mechanism of the influence of *B. subtilis* on aerobic stability has not been explored in sufficient depth in previous studies.

The next generation sequencing method enabled us to identify the microbial taxa associated with silage (Bai et al., 2022b). We hypothesized that inoculation with *L. buchneri* or *B. subtilis* could improve aerobic stability by altering the microbial community structure. Therefore, the aim of this study was to investigate the effects of *B. subtilis* or *L. buchneri* on the fermentation quality, aerobic stability, and bacterial and fungal communities of whole plant corn silage during aerobic exposure.

## 2. Materials and methods

### 2.1. Silage preparation and aerobic stability

The experimental planting area is located in Shenhe District, Shenyang City, Liaoning Province, Baicaoyuan Scientific Research Base of Shenyang Agricultural University (41^°^50’N, 123^°^34’E). The annual average temperature is 8.1^°^C, the annual average precipitation is 721.9 mm, the annual frost-free period is 150–170 days, and the soil type is brown earth loam containing 48 sand, 29% silt and 23% clay in the depth of 0–20 cm. The whole plant corn variety is Dongdan60, which is harvested at the wax maturity stage, and the stubble height is 15 cm. Whole plant corn was chopped to a length of approximately 1 cm by a grass shredding machine (Donghong No. 1, Donghong Mechanical Equipment Co., Ltd., Donggang, China), and 600 g of chopped material was applied to the (1) distilled sterile water control (CK), (2) *Lentilactobacillus buchneri* (Lallemand Animal Nutrition, Milwaukee, WI) (LB, 2 × 10^5^ cfu/g), and (3) *Bacillus subtilis* groups (Shandong Vland Biological Technology Co. LTD) (BS, 2 × 10^5^ cfu/g). The inoculant was dissolved in 3 mL sterile water and sprayed evenly. After thoroughly mixing, the materials were ensiled in vacuum-sealed silage bags (35 × 24 cm) and fermented at room temperature (23–28^°^C).

After 42 days of ensiling, silage bags were opened, removed approximately 200 g of the silage sample from the silage bag and add it to a beaker with a 250 mL volume, and aerobic stability was observed for 100 h. Beakers were then placed inside a Styrofoam block, and the silage samples were exposed to air at ambient temperature. Samples were taked at three time points of 0 h (AS0), 18 h (AS1), and 60 h (AS2) to analyse the fermentation quality and aerobic stability. The temperature during aerobic exposure was measured by a 64-well thermograph with a probe placed in the centre of the silage, with temperature changes recorded every 10 min (Zhao et al., 2021).

### 2.2. Analyses of chemical components and fermentation characteristics

Ten grams of silage sample was added to 90 mL of distilled water, and placed in a refrigerator at 4^°^C for 12 h. A pH metre with a glass electrode was used (PB-10, Sartorius Group, Göttingen, Germany) to measure the pH of the extract (Han et al., 2015). The leaching solution was filtered through a 0.22 μm filter membrane, and the contents of lactic acid (LA), acetic acid (AA), butyric acid (BA) and ethanol in the leaching solution were determined by high-performance liquid chromatography (HPLC; Waters1525, Waters Corporation, Milford, MA, United States). The analysis conditions were as follows: chromatographic column, Carbomix H-NP5 (Sepax Technologies, Inc., Newark, DE, United States); detector, refractive index detector (Waters2414, Waters Corporation, Milford, MA, United States); mobile phase, 2.5 mmol/L H_2_SO_4_; flow rate, 0.6 ml/min; column temperature, 55^°^C; sample volume, 10 μL (Wang et al., 2021). The organic acid concentrations were obtained by comparing the curves of the silage extracts with the curves of experimental standard. Ammonia nitrogen (AN) was determined by the phenol-sodium hypochlorite colorimetric method (Ke et al., 2017).

Approximately 100 g of silage was weighed, placed in a paper bag and dried in an oven for 48 h. The weight loss of the silage sample was recorded during the process, and the dry matter (DM) content of the sample was calculated. Water-soluble carbohydrates (WSC) were determined by anthrone-sulfuric acid colorimetry (Thomas, 1977), starch was determined by acid hydrolysis (Bai et al., 2022b), and crude protein (CP) was determined by the Kjeldahl method (Huang et al., 2021) using an autoanalyzer (Kjeltec 8400; FOSS Co. Ltd., Hillerød, Denmark). NDF (neutral detergent fiber assayed with a heat stable amylase; aNDF) and ADF (acid detergent fiber) were measured according to the Van Soest method (Van Soest et al., 1991) using an Ankom 2000 fiber analyser (Ankom, Macedon, NY, United States). The quantities of lactic acid bacteria, coliform bacteria, yeasts and filamentous fungi in silage were determined by culturing on MRS agar, violet-red bile agar and rose Bengal medium, respectively (Bai et al., 2022a).

### 2.3. Bacterial and fungal communities

Fresh samples (10 g) were put into 90 mL of sterile distilled water, shaken using a cryogenic oscillator (THZ-98C, Shanghai Yiheng Scientific Instrument Co. Ltd., Shanghai, China) at 4^°^C and 180 rpm for 30 min, and then filtered through three layers of sterile gauze. The filtrate was centrifuged in a cryogenic centrifuge (ST 16R, Thermo Fisher Scientific, Inc., Waltham, MA, USA) at 13,000 × g for 15 min at 4^°^C to enrich the sediment. Sediments were used for high-throughput sequencing (Wang et al., 2018).

Total DNA was extracted from bacteria and fungi in the silage samples with an E.Z.N.A. ^®^ Stool DNA Kit (Omega Bio-Tek, United States). The hypervariable region V3-V4 of full-length bacterial 16S rRNA genes were amplified by PCR with specific forward 341F (CCTAYGGGRBGCASCAG) and reverse 806R (GGACTACNNGGGTATCTAAT) primers and full-length fungal internal transcribed spacers (ITS) were amplified with specific forward ITS1F (CTTGGTCATTTAGAGGAAGTAA) and reverse ITS2R (GCTGCGTTCTTCATCGATGC) primers (Bai et al., 2021). The AxyPrep DNA Gel Extraction Kit (Axygen Biosciences, Union City, CA, United States) was used to purify the PCR products, and a Quantus™ Fluorometer (Promega, United States) was used for quantitative determination, using the MiSeq PE300 platform of the Illumina Company for sequencing (Shanghai Majorbio Bio-Pharm Technology Co. Ltd.). Next fastp and FLASH software programs were used to control the quality and splice raw sequences, respectively. Furthermore, UPARSE software was used to cluster sequences with 97% similarity in operational taxonomic units (OTUs). Using mothur software^1^ to calculate the alpha diversity, Chao, Shannon indices, etc., PCoA analysis (principal coordinate analysis) based on the Bray-Curtis distance algorithm was used to test the similarity of microbial community structure among samples. Species were selected for correlation heatmap graph analysis based on Spearman correlation | r| > 0.6 *P* < 0.05 (Wang et al., 2021). The sequencing data were submitted to the NCBI Sequence Read Archive database (accession: PRJNA890780).

### 2.4. Data statistics and analysis

Microbiological data were log10-transformed on a fresh basis. Two-way ANOVA was performed on the data using SPSS 22.0 to determine the effect of silage time and mixed silage ratio. The data for pH and organic acids were analysed using the general linear model procedure of the Statistical Package for Social Science (SPSS 21.0, SPSS, Inc., Chicago, IL, USA) according to the model:


$$Y_{ij}=\mu +T_{i}+D_{j}+{\left(T\times D\right)}_{ij}+\varepsilon_{ij}$$

where Y_*ij*_ represents the response variable, μ is the overall mean, T_*i*_ is the effect of inoculants, D_*j*_ is the effect of ensiling time, (T × D)_*ij*_ is the effect of the interaction between the inoculants and ensiling time, and ε_*ij*_ is the random residual error. Multiple comparisons were performed using Tukey’s method, and *P* < 0.05 indicated a significant difference.

## 3. Results

### 3.1. Characteristics of whole plant corn before ensiling

The contents of DM, pH, CP, WSC, and starch in whole plant corn fresh forage were 342.84 g/kg FW, 5.28, 71.86, 150.95, and 326.26 g/kg DM, respectively, and the coliform bacteria, lactic acid bacteria, and yeasts were 5.55, 6.00, and 6.42 lg CFU/g, respectively. The number of filamentous fungi was lower than the detection level (Table 1).

**TABLE 1:** Chemical composition of whole plant corn before ensiling.

Item	Whole plant corn	SEM
DM (g/kg FW)	342.84	0.646
pH	5.28	0.025
CP (g/kg DM)	71.86	0.055
aNDF (g/kg DM)	420.31	1.255
ADF (g/kg DM)	211.10	0.637
Starch (g/kg DM)	326.26	0.896
WSC (g/kg DM)	150.95	0.387
Lactic acid bacteria (lg CFU/g)	6.00	0.028
Yeasts (lg CFU/g)	6.42	0.017
Filamentous fungi (lg CFU/g)	<2.00	–

FW, fresh weight; DM, dry matter; CP, crude protein; aNDF, neutral detergent fiber assayed with a heat stable amylase; ADF, acid detergent fibre; WSC, water soluble carbohydrates; SEM, standard error of the mean.

### 3.2. Fermentation quality, nutrient composition and microbial counts of silage

The pH, AA and AN contents of the BS and LB groups were higher than those of the CK group (*P* < 0.05; Table 2), the LA concentration of the LB group was higher than that of the other two groups (*P* < 0.05), and BA was not detected in any of the groups (the minimum detectable limit is approximately 0.1 μg/mL). The CP content of the BS group was lower than that of the CK group (*P* < 0.05), the WSC content in the LB group was higher than those in the other two groups (*P* < 0.05), and there was no significant difference in the contents of aNDF and ADF among the groups (*P* > 0.05). The numbers of lactic acid bacteria in the BS group were significantly higher than those in the CK and LB groups (*P* < 0.05). The number of filamentous fungi in each group was lower than the detection level (<2.00 lg CFU/g).

**TABLE 2:** Fermentation quality, nutrient composition and microbial counts of whole plant corn silage after 42 days of ensiling.

Item	Treatment			SEM	*P*-value
	CK	LB	BS		
pH	3.50c	3.57b	3.63a	0.006	<0.01
LA (g/kg DM)	38.87b	49.70a	38.13b	0.147	<0.05
AA (g/kg DM)	4.50b	6.70a	6.40a	0.026	<0.05
BA (g/kg DM)	-	-	-	-	-
Ethanol (g/kg DM)	12.57a	7.00b	2.60c	0.054	<0.01
AN (TN%)	2.40b	3.28a	3.73a	0.142	<0.05
DM (g/kg FW)	368.86	368.43	355.68	0.380	0.303
CP (g/kg DM)	83.52a	80.94ab	77.95b	0.052	<0.05
aNDF (g/kg DM)	396.69	396.15	385.36	0.700	0.766
ADF (g/kg DM)	218.23	202.12	207.71	0.621	0.608
Starch (g/kg DM)	350.43a	300.36b	340.51ab	0.957	0.157
WSC (g/kg DM)	18.56b	37.48a	17.96b	0.175	<0.01
Lactic acid bacteria (lg CFU/g)	6.38b	6.35b	7.09a	0.128	0.065
Yeasts (lg CFU/g)	3.68	3.86	3.91	0.087	0.930
Filamentous fungi (lg CFU/g)	<2.00	<2.00	<2.00	-	-

Means with different lowercase letters in the same row (a–c) differ significantly (*P* < 0.05). LA, lactic acid; AA, acetic acid; BA, butyric acid; AN, ammonia nitrogen; TN, total nitrogen. FW, fresh weight; DM, dry matter; CP, crude protein; aNDF, neutral detergent fiber assayed with a heat stable amylase; ADF, acid detergent fibre; WSC, water soluble carbohydrates; CK, control, no inoculations; LB; silages inoculated with *Lentilactobacillus buchneri*; BS, silages inoculated with *Bacillus subtilis*; SEM, standard error of the mean.

### 3.3. Dynamics of temperature and fermentation quality during aerobic exposure

**FIGURE 1:**
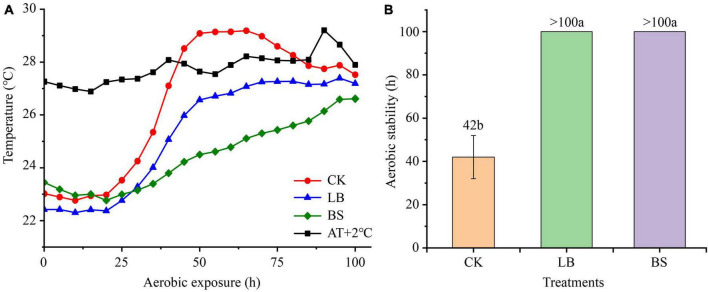
Dynamics of whole plant corn silage temperature (^°^C) versus ambient temperature during aerobic exposure **(A)** and the time when silage temperature was 2^°^C below ambient temperature **(B)**. Means with different lowercase letters (a–b) indicate significant differences in aerobic stability time (*P* < 0.05). AT + 2^°^C, ambient temperature +2^°^C. CK, control, no inoculations; LB, silages inoculated with *Lentilactobacillus buchneri*; BS, silages inoculated with *Bacillus subtilis*.

**FIGURE 2:**
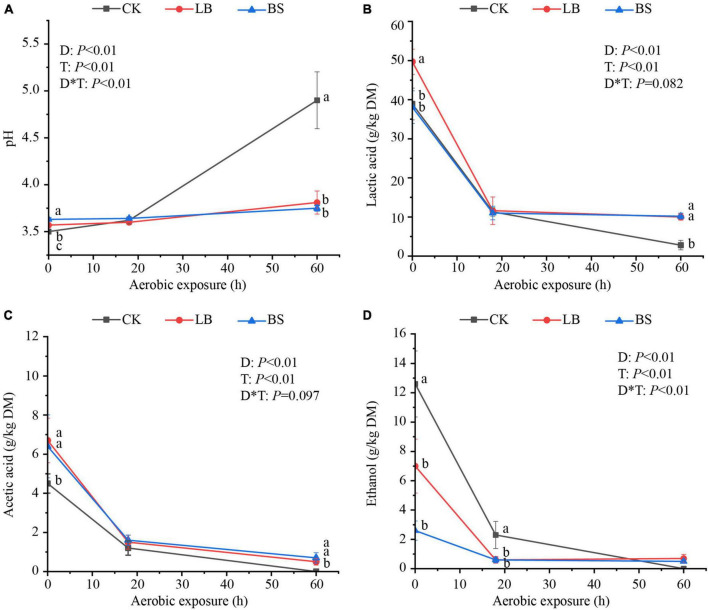
Fermentation quality (**A**, pH; **B**, lactic acid; **C**, acetic acid; **D**, ethanol) of whole plant corn silage during aerobic exposure. Means with different lowercase letters (a–c) indicate significant differences in different treatments at the same time (*P* < 0.05). D, Aerobic exposure time; T, treatment; D*T, interaction between aerobic exposure time and treatment; CK, control; no inoculations; LB, silages inoculated with *Lentilactobacillus buchneri*; BS, silages inoculated with *Bacillus subtilis*.

The aerobic stability levels of the LB and BS treatment groups were better than that of the CK group (Figure 1). Aerobic exposure time and inoculant treatment affected pH, LA, AA, and ethanol content (*P* < 0.05; Figure 2), and their interactions affected pH and ethanol content (*P* < 0.05). With the prolongation of aerobic exposure time, the pH value of each group gradually increased, the contents of LA, AA, and ethanol gradually decreased, and the pH of CK increased the most, from 3.52 to 4.90. LA and AA in the LB and BS groups were higher than those in the CK group at AS2 (*P* < 0.05).

### 3.4. Microbial community during aerobic exposure

**FIGURE 3:**
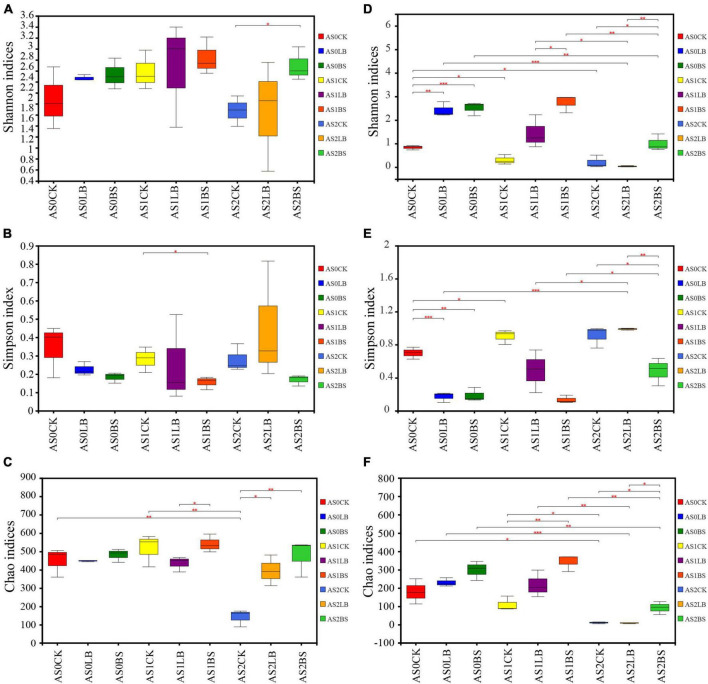
Differences in bacterial **(A–C)** and fungal **(D–F)** community diversity and richness in whole plant corn silage during aerobic exposure. **(A)** Shannon index. **(B)** Simpson index. **(C)** Chao index. **(D)** Shannon index. **(E)** Simpson index. **(F)** Chao index. *Significance at *P* < 0.05; **significance at *P* < 0.01; ***significance at *P* < 0.001. AS0CK, not inoculated silage at 0 h of aerobic exposure; AS0LB, *Lentilactobacillus buchneri* inoculated silage at 0 h of aerobic exposure; AS0BS, inoculated *Bacillus subtilis* silage at 0 h of aerobic exposure; AS1CK, not inoculated silage at 18 h of aerobic exposure; AS1LB, *Lentilactobacillus buchneri* inoculated silage at 18 h of aerobic exposure; AS1BS, inoculated Bacillus subtilis silage at 18 h of aerobic exposure; AS2CK, not inoculated silage at 60 h of aerobic exposure; AS2LB, *Lentilactobacillus buchneri* inoculated silage at 60 h of aerobic exposure; AS2BS, inoculated *Bacillus subtilis* silage at 60 h of aerobic exposure.

**FIGURE 4:**
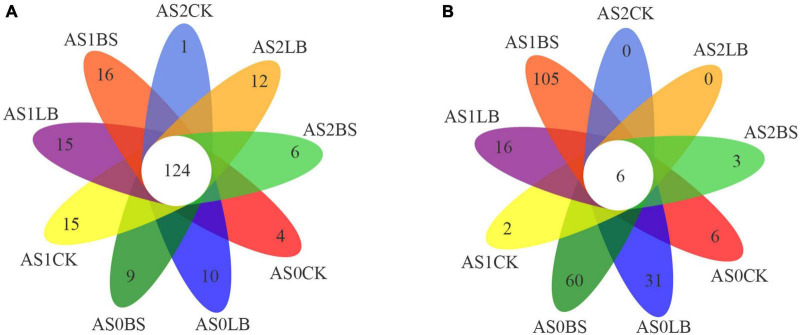
Venn diagram of bacterial **(A)** and fungal **(B)** operational taxonomic units (OTUs) in whole plant corn silage during aerobic exposure. AS0CK, not inoculated silage at 0 h of aerobic exposure; AS0LB, *Lentilactobacillus buchneri* inoculated silage at 0 h of aerobic exposure; AS0BS, inoculated *Bacillus subtilis* silage at 0 h of aerobic exposure; AS1CK, not inoculated silage at 18 h of aerobic exposure; AS1LB, *Lentilactobacillus buchneri* inoculated silage at 18 h of aerobic exposure; AS1BS, inoculated *Bacillus subtilis* silage at 18 h of aerobic exposure; AS2CK, not inoculated silage at 60 h of aerobic exposure; AS2LB, *Lentilactobacillus buchneri* inoculated silage at 60 h of aerobic exposure; AS2BS, inoculated *Bacillus subtilis* silage at 60 h of aerobic exposure.

The Chao index of the CK group of bacteria decreased with increasing aerobic exposure time (*P* < 0.05; Figures 3A–C), and the Shannon and Simpson indices were not significantly different (*P* > 0.05). At AS2, the Chao index was higher in the LB group than in the CK group (*P* < 0.05), and the Chao and Shannon indices were higher in the BS group than in the CK group (*P* < 0.05). The Shannon and Chao indices of the fungal communities gradually decreased with increasing aerobic exposure time (*P* < 0.05 Figures 3D–F). The Shannon and Chao indices of the BS group were higher than those of the other two groups at AS1 and AS2 (*P* < 0.05), and the Simpson index was lower than those of the other two groups at AS1 (*P* < 0.05).

operational taxonomic unit clustering of the sequences according to a similarity of 97% (Figure 4), revealed 124 shared OTUs among the treated bacterial samples, and there were more OTUs unique to BS under aerobic exposure to AS1 (Figure 4A). There were 6 shared OTUs in all treated sample fungi, and aerobic exposure to the AS0, AS1, and BS groups had more OTUs during aerobic exposure, up to 105 at AS1 (Figure 4B).

**FIGURE 5:**
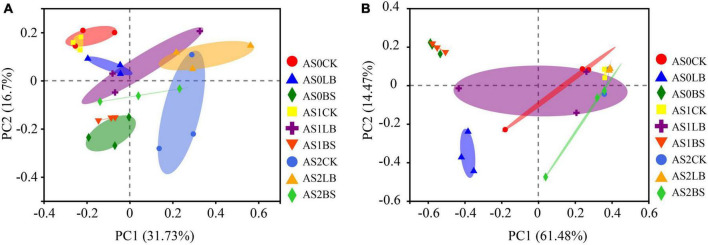
Principal component analysis of bacterial communities **(A)** and fungal **(B)** operational taxonomic units (OTUs) in whole plant corn silage during aerobic exposure. AS0CK, not inoculated silage at 0 h of aerobic exposure; AS0LB, *Lentilactobacillus buchneri* inoculated silage at 0 h of aerobic exposure; AS0BS, inoculated *Bacillus subtilis* silage at 0 h of aerobic exposure; AS1CK, not inoculated silage at 18 h of aerobic exposure; AS1LB, *Lentilactobacillus buchneri* inoculated silage at 18 h of aerobic exposure; AS1BS, inoculated *Bacillus subtilis* silage at 18 h of aerobic exposure; AS2CK, not inoculated silage at 60 h of aerobic exposure; AS2LB, *Lentilactobacillus buchneri* inoculated silage at 60 h of aerobic exposure; AS2BS, inoculated *Bacillus subtilis* silage at 60 h of aerobic exposure.

The LB and BS groups were separated from the CK group in the bacterial community at AS0 and AS1 (Figure 5A). Components 1 and 2 could be well-explained in fungal community structure, and the LB and BS groups were separated from the CK group at AS0 (Figure 5B).

**FIGURE 6:**
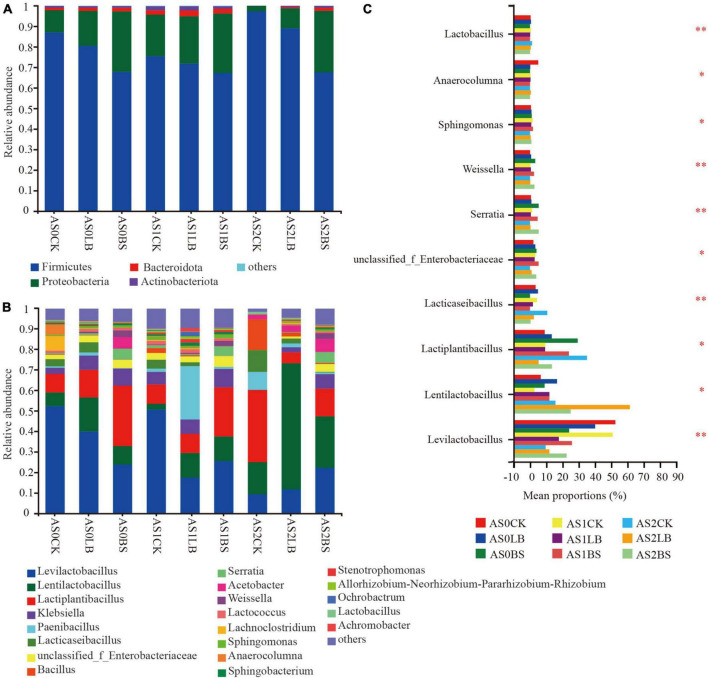
Dynamic changes in relative abundance of bacteria at the phylum level **(A)** and genus level **(B)** during aerobic exposure and the differences in genus level **(C)**. *Significance at *P* < 0.05; ^**^significance at *P* < 0.01. AS0CK, not inoculated silage at 0 h of aerobic exposure; AS0LB, *Lentilactobacillus buchneri* inoculated silage at 0 h of aerobic exposure; AS0BS, inoculated *Bacillus subtilis* silage at 0 h of aerobic exposure; AS1CK, not inoculated silage at 18 h of aerobic exposure; AS1LB, *Lentilactobacillus buchneri* inoculated silage at 18 h of aerobic exposure; AS1BS, inoculated *Bacillus subtilis* silage at 18 h of aerobic exposure; AS2CK, not inoculated silage at 60 h of aerobic exposure; AS2LB, *Lentilactobacillus buchneri* inoculated silage at 60 h of aerobic exposure; AS2BS, inoculated *Bacillus subtilis* silage at 60 h of aerobic exposure.

Whole plant corn silage was mainly regulated by *Firmicutes* and *Proteobacteria* during aerobic exposure, and *Firmicutes* had the highest relative abundance (Figure 6A). From AS0 to AS1 of aerobic exposure, the relative abundance of *Proteobacteria* increased and that of *Firmicutes* decreased in each treatment. At AS2, the relative abundances of *Proteobacteria* in the CK and LB groups decreased, but the BS group remained unchanged. The dominant genera were *Levilactobacillus*, *Lentilactobacillus* and *Lactiplantibacillus* (Figure 6B). During the aerobic exposure, differences were observed among treatments in *Levilactobacillus*, *Lentilactobacillus*, *Lactiplantibacillus*, *unclassified_f_Enterobacteriaceae*, *Serratia*, *Weissella*, etc., (*P* < 0.05; Figure 6C). At AS0, the relative abundance of *Serratia* in the BS group was higher than those in the CK and LB groups (*P* < 0.05), and the relative abundance of *Lentilactobacillus* in the LB and BS group were lower than that in the CK group at AS2 (*P* < 0.05).

**FIGURE 7:**
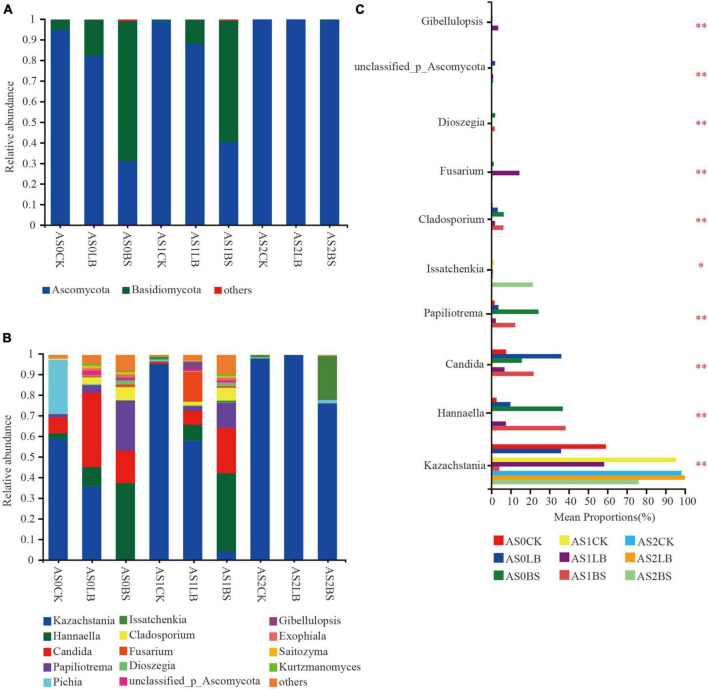
Dynamic changes in relative abundance of fungi at the phylum level **(A)** and genus level **(B)** during aerobic exposure and the differences in genus level **(C)**. *Significance at *P* < 0.05; ^**^significance at *P* < 0.01. AS0CK, not inoculated silage at 0 h of aerobic exposure; AS0LB, *Lentilactobacillus buchneri* inoculated silage at 0 h of aerobic exposure; AS0BS, inoculated *Bacillus subtilis* silage at 0 h of aerobic exposure; AS1CK, not inoculated silage at 18 h of aerobic exposure; AS1LB, *Lentilactobacillus buchneri* inoculated silage at 18 h of aerobic exposure; AS1BS, inoculated *Bacillus subtilis* silage at 18 h of aerobic exposure; AS2CK, not inoculated silage at 60 h of aerobic exposure; AS2LB, *Lentilactobacillus buchneri* inoculated silage at 60 h of aerobic exposure; AS2BS, inoculated *Bacillus subtilis* silage at 60 h of aerobic exposure.

The dominant fungi during aerobic exposure of whole plant corn silage were *Ascomycota* and *Basidiomycota* (Figure 7A). The relative abundance of *Basidiomycota* in each treatment increased with prolonged aerobic exposure, but the relative abundance of *Basidiomycota* in the LB and BS groups were lower than those in the CK group at AS0 and AS1, delaying the trend of increasing *Basidiomycota* relative abundance. The composition of fungal species at the genus level is shown in Figure 7B. The relative abundance of *Kazachstania* gradually increased during aerobic exposure, but the relative abundance of *Kazachstania* in the LB and BS groups were lower than those in the CK group at AS0 and AS1. At AS0, the CK group was dominated by fungi such as *Kazachstania* and *Pichia*, *Candida* accounted for a large proportion in the LB treatment, the relative abundances of *Papiliotrema* and *Hannaella* in the BS group accounted for the largest proportions, and the relative abundance of *Issatchenkia* in the BS group was higher than those in the other treatments at AS2 (*P* < 0.05; Figure 7C).

**FIGURE 8:**
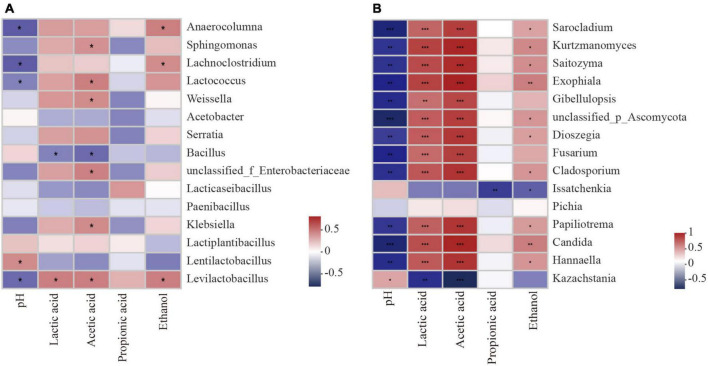
Spearman analysis between silage parameters and bacterial species **(A)** fungal species **(B)** during aerobic exposure. *Significance at *P* < 0.05; **significance at *P* < 0.01; ***significance at *P* < 0.001.

Silage pH was negatively correlated with the abundances of *Levilactobacillus*, *Lactococcus*, etc., in the bacterial community (*P* < 0.05; Figure 8A). The contents of LA and AA were positively correlated with the abundance of *Levilactobacillus* (*P* < 0.05) but negatively correlated with the abundance of *Bacillus* (*P* < 0.05). From the correlation analysis of fungal and fermentation characteristics during aerobic exposure, the pH value was negatively correlated with the abundance of *Hannaella*, *Candida*, etc., in the fungal community (*P* < 0.01; Figure 8B), but positively correlated with the abundance of *Kazachstania* (*P* < 0.05). The contents of LA and AA were positively correlated with the abundance of *Hannaella*, *Candida*, etc., (*P* < 0.01), but negatively correlated with the abundance of *Kazachstania* (*P* < 0.01).

**FIGURE 9:**
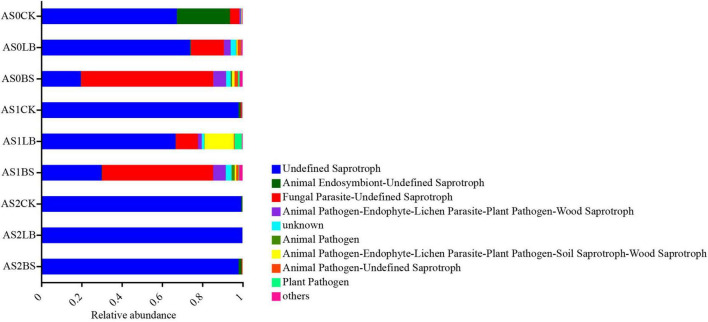
Variations in composition of fungal functional groups inferred by FUNGuild during aerobic exposure. AS0CK, not inoculated silage at 0 h of aerobic exposure; AS0LB, *Lentilactobacillus buchneri* inoculated silage at 0 h of aerobic exposure; AS0BS, inoculated *Bacillus subtilis* silage at 0 h of aerobic exposure; AS1CK, not inoculated silage at 18 h of aerobic exposure; AS1LB, *Lentilactobacillus buchneri* inoculated silage at 18 h of aerobic exposure; AS1BS, inoculated *Bacillus subtilis* silage at 18 h of aerobic exposure; AS2CK, not inoculated silage at 60 h of aerobic exposure; AS2LB, *Lentilactobacillus buchneri* inoculated silage at 60 h of aerobic exposure; AS2BS, inoculated *Bacillus subtilis* silage at 60 h of aerobic exposure.

Functional predictions of fungal communities were based on FUNGuild (Figure 9) determined that the undefined saprotroph was inhibited in the LB and BS groups compared to AS1CK, which became dominant in all treatments at AS2.

## 4. Discussion

### 4.1. Material characteristics and silage quality

The composition of the fresh forage in this experiment was within the expected range for whole plant corn (Ranjit and Kung, 2000; Ferraretto and Shaver, 2015). It is generally believed that pH and AN contents lower than 4.2 and 10% TN indicate good silage quality (Ahmadi et al., 2019). In the quality analysis of silage after 42 days of fermentation, the pH value and AN of the LB and BS treatments were higher than the CK group, but both were within a good range, and the ethanol content was low with no BA production (Sifeeldein et al., 2019), indicating that the fermentation quality of all treatments was in good condition. The LA content of the LB group was higher than that of the CK group, presumably because of the higher abundance of *Lentilactobacillus* in the LB group (Wu et al., 2022b). *Lentilactobacillus buchneri* can convert part of the lactic acid from the silage to acetic acid, increasing the acetic acid concentration of silage and causing small increases in pH (Schmidt and Kung, 2010; Benjamim da Silva et al., 2021; Ferrero et al., 2021b). In this trial, LB inoculation increased the AA content, resulting in an increase in pH value. The AA content of the BS group was significantly higher than that of the CK group, which may account for the better aerobic stability of the BS group (Basso et al., 2012; Bai et al., 2022a). Acetic acid has good antifungal properties and can inhibit the growth of fungi, thereby inhibiting the process of aerobic spoilage of silage (Santos et al., 2020). There was no significant difference in the number of yeasts among treatments, and the number of filamentous fungi was below the detection limit, because the lower pH maintains an acidic environment and inhibits the growth of yeasts and filamentous fungi (Sun et al., 2021).

The relative abundances of *unclassified_f_Enterobacteriaceae* and *Klebsiella* in the BS treatment were higher, with a higher pH, presumably explaining the higher protease activity during silage, which may account for the higher CP degradation (Chen et al., 2014). The study by Bernardi et al. (2019) in the literature also observed a decrease in the CP content of silage after the addition of BS with an increasing concentration of addition, similar to the results of this experiment. The content of aNDF and ADF in BS group did not differ, indicating that *B. subtilis* has the low fiber dissolving activity in this experiment (Guo et al., 2022). In the LB group, the WSC content was higher and the starch content was lower than in the CK group, which is similar to the results of Comino et al. (2014).

### 4.2. Aerobic stability of silage

Usually, the core temperature of silage is 2^°^C higher than the ambient temperature to measure whether aerobic deterioration occurs (Ranjit and Kung, 2000). In this study, the aerobic stabilization times were higher in the LB and BS groups than in the CK group, suggesting that inoculation with LB or BS improves aerobic stability. This is due to the higher AA concentrations in the LB and BS groups at AS0, inhibiting the growth of aerobic microorganisms (Basso et al., 2012). In addition, BS can produce antifungal and antibacterial substances that inhibit the growth of undesirable microorganisms such as yeast (Bonaldi et al., 2021).

In the present study, all treatments of whole plant corn silage significantly increased the pH and decreased the LA and AA contents with prolonged aerobic exposure, because in the process of aerobic exposure, aerobic microorganisms decompose the organic acids such as lactic acid and acetic acid produced in the process of ensilage, and produced carbon dioxide (CO_2_) and water, which leads to an increase in the pH value (Tabacco et al., 2011; Li et al., 2017; Guo et al., 2022). Compared to the CK group, the LB or BS groups showed slower increases in pH and high LA and AA residues, indicating that the addition of LB and BS retarded the loss of material from the silage during aerobic exposure, in line with the findings of Lara et al. (2016). In the study of Yuan et al. (2018), ethanol as an exogenous addition (25 ml/kg FW) had the potential to inhibit aerobic spoilage. The ethanol in this experiment was a product of natural fermentation. The ethanol content of the CK group, although higher than those of the LB and BS groups, was well below the level of exogenous addition, which may account for the lack of effect on aerobic stability (Huisden et al., 2009).

### 4.3. Microbial community in silage

Our results showed that the duration of aerobic exposure had no significant effect on the Shannon and Simpson indices of the whole plant corn silage bacterial community, indicating that bacterial diversity was maintained in silage during the aerobic exposure phase (Drouin et al., 2019). In contrast, bacterial richness was reduced in the CK group in terms of the Chao index; presumably, the higher pH in the later stages of aerobic exposure provided conditions for the proliferation of spoilage microorganisms, which in turn reduced bacterial richness (Wu et al., 2022a). Inoculation with LB or BS maintained bacterial diversity and richness better during aerobic exposure. Principal component analysis clearly showed the differences between groups of bacterial and fungal communities, respectively, indicating that inoculation with LB or BS altered bacterial and fungal communities during aerobic exposure. Fungal diversity decreased in diversity and abundance with increasing exposure time, indicating that the fungal community composition gradually simplified (Zhang et al., 2019). The fungal community diversities of the LB and BS treatments were higher than that of the CK group, and the fungal abundance and special OTU numbers were higher in the BS of the silage inoculation group than in the CK and LB groups, which may account for the higher aerobic stability of LB or BS (Bai et al., 2020).

Similar to most studies, the bacterial communities of whole plant corn silage in all treatments were mainly regulated by *Firmicutes* and *Proteobacteria* during aerobic exposure in this experiment (Drouin et al., 2019; Liu et al., 2020). The inoculation of LB or BS significantly affected the relative abundances of *Levilactobacillus, Lentilactobacillus*, *Lactiplantibacillus*, *Lacticaseibacillus*, *Enterobacteria* and *Weissella*, etc. Regarding the genus *Lactobacillus*, to which *Lactiplantibacillus*, *Lentilactobacillus*, and *Lacticaseibacillus* originally belonged (Zheng et al., 2020), in the study of Wu et al. (2022a), inoculation with *L. buchneri* improved aerobic stability and maintained the high relative abundance of *Lactobacillus* during aerobic exposure. In this experiment, the relative abundance of *Lentilactobacillus* at AS2 was higher in the LB and BS groups than in the CK group, which may be one of the reasons for the improved aerobic stability of the LB and BS groups. Research has reported that *Enterobacteria* are able to improve aerobic stability (Wilkinson and Davies, 2013). We found that the relative abundance of *Enterobacteria* was significantly different among BS groups at AS2, which may be one of the reasons for its better aerobic stability. During aerobic exposure, the relative abundance of *Weissella* in the BS group was higher than those in the other two groups. *Weissella*, another major microorganism in all silage throughout the fermentation process (Guan et al., 2018), was able to produce lactate (Ni et al., 2018). In the significant differences in bacterial relative abundance analysis, *Serratia* was the most abundant genus in the BS group, *Serratia* can produce prodigiosin, a secondary metabolite that might inhibit the proliferation of fungi (Zhang et al., 2019), which may be one of the reasons for the higher aerobic stability in the BS group. *Bacillus* caused aerobic spoilage in a previous study (Liu et al., 2020). In this experiment, the relative abundance of *Bacillus* in the LB and BS groups were lower than that in the CK group at AS2, indicating that the addition of LB and BS could inhibit the growth of *Bacillus* and improve aerobic stability.

*Ascomycota* and *Basidiomycota* were the dominant fungal phyla of whole plant corn silage, which was similar to the findings of Liu et al. (2019). During aerobic exposure, LB or BS inoculation delayed the increasing trend of the relative abundance of *Basidiomycota*, indicating that inoculation with LB or BS significantly altered the structure of the whole plant corn silage fungal population during aerobic exposure (Zhang et al., 2019). Wang et al. (2021) found that *Kazachstania* was dominant in corn silage with poor aerobic stability. In the early stage of aerobic exposure, *Kazachstania*, *Hannaella*, and *Candida* possessed dominant positions in the fungal community; as time progressed, the relative abundance of *Kazachstania* gradually increased, becoming all the dominant fungi in AS2. The inoculation of LB or BS delayed fungal succession and inhibited *Kazachstania*, thus resulting in higher aerobic stability. At AS0, the relative abundance of *Candida* was significantly higher in the LB group than in the other two groups, and the relative abundances of *Hannaella* were significantly higher in the BS groups of AS1 and AS2. *Candida* and *Hannaella* were the fungal genera related to aerobic spoilage (Drouin et al., 2019; Wang et al., 2020), which may play a role in the early stage of aerobic exposure in this experiment but is not the decisive factor triggering aerobic spoilage. The community composition of the BS group did not change much at AS1 and AS2, but the relative abundance of *Issatchenkia* was higher in BS at AS2, and *Issatchenkia* also dominated after aerobic exposure of barley silage, as reported by Liu et al. (2020).

The FUNGuild prediction results showed that, inoculation with LB and BS reduced the relative abundance of undefined saprotroph and increased the relative abundance of fungal parasite-undefined saprotroph during the aerobic exposure phase, presumably associated with increased aerobic stability, undefined saprotroph was dominated by the highest relative abundance of undefined saprotroph in each treatment after aerobic decay (Wu et al., 2022c).

## 5. Conclusion

Compared to the CK group, LB or BS inoculated in whole plant corn silage maintained satisfactory fermentation quality, improved aerobic stability, and effectively inhibited aerobic spoilage. In combination with the analysis of the microbial community structure, it was found that the delay in aerobic spoilage may be related to the *Bacillus* and *Kazachstania* inhibited and higher relative abundance of fungal parasite-undefined saprotroph by inoculated of LB or BS.

## Data availability statement

The datasets presented in this study can be found in online repositories. The names of the repository/repositories and accession number(s) can be found in the article/supplementary material.

## Author contributions

HY, JS, ZY, CB, and YX designed the study and wrote the manuscript. HY, MZ, GP, HZ, and RY performed the experiments. JS, ZY, CB, and YX reviewed and edited the manuscript. HY, MZ, and GP analyzed the data. ZY, CB, and YX funded and supervised the experiments. All authors reviewed the manuscript and approved the submitted version.

## Footnotes

1
http://www.mothur.org/wiki/Calculators
